# Optimisation of biomass, exopolysaccharide and intracellular polysaccharide production from the mycelium of an identified *Ganoderma lucidum* strain QRS 5120 using response surface methodology

**DOI:** 10.3934/microbiol.2019.1.19

**Published:** 2019-01-22

**Authors:** Sugenendran Supramani, Rahayu Ahmad, Zul Ilham, Mohamad Suffian Mohamad Annuar, Anita Klaus, Wan Abd Al Qadr Imad Wan-Mohtar

**Affiliations:** 1 Functional Omics and Bioprocess Development Laboratory, Institute of Biological Sciences, Faculty of Science, University of Malaya, 50603, Kuala Lumpur, Malaysia; 2 Halal Action Laboratory, Kolej Permata Insan, University Sains Islam Malaysia, Bandar Baru Nilai, 71800, Nilai, Negeri Sembilan, Malaysia; 3 Institute of Biological Sciences, Faculty of Science, University of Malaya, 50603, Kuala Lumpur, Malaysia; 4 Institute for Food Technology and Biochemistry, Faculty of Agriculture, University of Belgrade, Nemanjina 6, 11080 Belgrade, Serbia

**Keywords:** *Ganoderma lucidum*, response surface methodology, submerged-liquid fermentation, exopolysaccharide, intracellular polysaccharide

## Abstract

Wild-cultivated medicinal mushroom *Ganoderma lucidum* was morphologically identified and sequenced using phylogenetic software. In submerged-liquid fermentation (SLF), biomass, exopolysaccharide (EPS) and intracellular polysaccharide (IPS) production of the identified *G.* *lucidum* was optimised based on initial pH, starting glucose concentration and agitation rate parameters using response surface methodology (RSM). Molecularly, the *G. lucidum* strain QRS 5120 generated 637 base pairs, which was commensurate with related *Ganoderma* species. In RSM, by applying central composite design (CCD), a polynomial model was fitted to the experimental data and was found to be significant in all parameters investigated. The strongest effect (*p* < 0.0001) was observed for initial pH for biomass, EPS and IPS production, while agitation showed a significant value (*p* < 0.005) for biomass. By applying the optimized conditions, the model was validated and generated 5.12 g/L of biomass (initial pH 4.01, 32.09 g/L of glucose and 102 rpm), 2.49 g/L EPS (initial pH 4, 24.25 g/L of glucose and 110 rpm) and 1.52 g/L of IPS (and initial pH 4, 40.43 g/L of glucose, 103 rpm) in 500 mL shake flask fermentation. The optimized parameters can be upscaled for efficient biomass, EPS and IPS production using *G. lucidum*.

## 1. Introduction

*Ganoderma lucidum* is a mushroom traditionally used in Chinese medicine for the prevention and treatment of human disease. Studies on *G. lucidum* and its products have reported beneficial biological, health-preserving and therapeutic effects [1]–[5]. Fungal polysaccharide has been shown to possess antioxidant, anti-inflammatory, antibacterial, antifungal and antiviral activities [4],[6]–[10], and can be obtained *via* solid substrate fermentation (SSF) or submerged-liquid fermentation (SLF). However, owing to the inherent nature of the solid substrate in SSF, fungal growth occurs through mycelial colonization of the substrate bed [11]. Furthermore, poor mass transfer and heterogeneity issues within solid matrix render polysaccharide production in SSF a highly time-consuming method. SLF has been shown to be superior to SSF in this respect [11],[12].

In SLF, a suspended biomass grows as a cluster of mycelia that eventually stabilize to form pellets [13] in the form of densely branched hyphae forming a compact ovoid shape. Fungal polysaccharide exists in two forms, exopolysaccharide (EPS) and intracellular polysaccharide (IPS). EPS is secreted outside the mycelium whereas IPS is produced inside the mycelium [10],[14]. Generally, total polysaccharide content produced by the mushroom thus comprises both EPS and IPS. Many factors affect the cultivation of biomass and polysaccharide production in SLF, including pH, agitation speed, oxygen transfer rate (OTR), glucose concentration and temperature [15],[16].

Hence, to enhance the cultivation of biomass and polysaccharide production in SLF, where the key parameters interact with each other in a complex manner, response surface methodology (RSM) represents the most effective solution compared with the one-factor-at-a-time (OFAAT) method [15]. In this study, RSM was used to study the interaction and correlation between the set of experimental variables and obtained results, and to subsequently establish the optimised conditions. The medicinal mushroom *G. lucidum* was subjected to morphological and molecular analyses prior to liquid fermentation. Next, a preliminary study was conducted using the OFAAT method to obtain baseline data and the working ranges of the selected SLF parameters, prior to the optimisation of biomass, exopolysaccharide (EPS) and intracellular polysaccharide (IPS) production. The selected parameters were initial pH, glucose concentration and agitation rate.

## 2. Materials and methods

### 2.1. Molecular characterisation

#### 2.1.1. Mushroom mycelium

**Figure 1.**
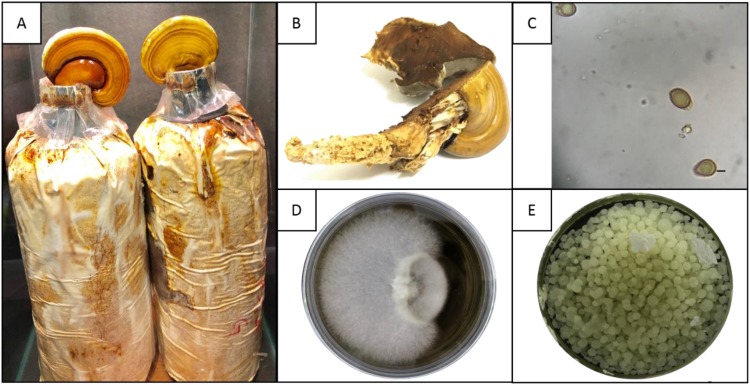
Different stages of *Ganoderma lucidum* QRS 5120 (A) obtained from Expo Hill, Mushroom Unit, University Putra Malaysia. (B) sliced fruiting body of *Ganoderma lcuidum* QRS 5120. (C) Basidiospores of *Ganoderma lucidum* QRS 5120 (Bar = 10 µm). (D) mycelium of *Ganoderma lucidum* QRS 5120 (Day 7). (E) pellets formation in submerged fermentation at day 7 (Bar = 0.05 cm).

The fruiting body of *Ganoderma lucidum* was obtained from the Mushroom Unit, Expo Hill, Universiti Putra Malaysia (UPM). The appearance and structure of the fruiting body (Figure 1A) and the basidiospores structure (Figure 1C) was first evaluated to validate the fungus. Next, with some modification of the Stamets [17] method, tissue culture was performed to obtain the mycelium. The fruiting body was washed with 99.9% ethanol (Sigma-Aldrich, Dorset, UK) for 10 s and dried in a laminar flow. Then, it was cracked using a scalpel and the inner part of the fruiting body was twisted and removed using forceps (Figure 1B). The tissue obtained was placed on malt extract agar (MEA) (Sigma-Aldrich, Dorset, UK) and maintained at room temperature until signs of mycelium growth were observed. The mycelium was then sub-cultured onto fresh MEA to obtain pure mycelium (Figure 1D), which was used as an initial culture for preservation in a potato dextrose agar (PDA) (Sigma-Aldrich, Dorset, UK) slant at 4 °C.

#### 2.1.2. Preparation of mycelium for DNA extraction

The mycelium was separated from agar and placed in pre-cooled pestle and ground to a fine powder under liquid nitrogen. The powder was freeze-dried and stored in an Eppendorf tube (Eppendorf no. 0030120973, Hamburg, Germany) at −20 °C [18],[19].

#### 2.1.3. *gDNA* extraction

The fine powdered mycelium (30 mg) was resuspended and lysed in lysis buffer (500 µL) by pipetting multiple times until the suspension became foamy. RNAase A (EN0531, Thermo Scientific, Waltham, MA, USA) was added and the mixture was incubated for 5 min at 37 °C. To remove the cell debris, polysaccharide and protein, NaCl_2_ solution (165 µL, 5 mol/L) was added and the tube was inverted multiple times before centrifugation (13,000 rpm, 20 min, 4 °C). The resulting supernatant was transferred to a fresh tube and mixed with chloroform (400 µL) and phenol (400 µL) by gentle inversion of the tube multiple times until the solution turned cloudy. The mixture was centrifuged (13,000 rpm, 20 min, 4 °C) and the aqueous phase was removed and extracted using an equal volume of chloroform. DNA was precipitated using 95% ethanol (2 volumes) and purified from polysaccharide by the addition of lysis buffer (500 µL) and mixing by gentle pipetting. NaCl (165 µL, 5 mol/L) was added and mixed by gentle inversion multiple times. To extract the purified DNA, chloroform (2 volumes) was added and the sample was centrifuged (13,000 rpm, 10 min, 4 °C). DNA was precipitated using ethanol (95%) and washed three times in ice-cold ethanol (70%). The washed DNA was dried, dissolved in Tris-EDTA buffer (50 µL) and stored at −20 °C [19].

#### 2.1.4. PCR amplification

DNA dissolved in TE buffer (Thermo Fisher no. 12090015, Invitrogen, Waltham, MA, USA) was brought to room temperature slowly from −20 °C. Using universal primers (ITS1 and ITS4), the fungal ITS gene was amplified. First, the solution (500 µL) was added to PCR tubes (Eppendorf no. 0030124332, Hamburg, Germany). Then, 0.5 pmol of ITS1 and ITS4 was added following by deoxynucleotide triphosphates (dNTPs, 200 μM each) (Promega no. U1511, Madison, OH, USA), 0.5 U DNA polymerase (Promega no. D1501, Madison, OH, USA), supplied PCR buffer (ThermoFisher no. 14966123, Platinum II Green PCR Buffer) and water. PCR was performed as follow: 1 cycle (98 °C for 2 min) for initial denaturation; 25 cycles (98 °C for 15 secs; 60 °C for 30 secs; 72 °C for 30 sec) for annealing and extension, and 1 cycle (72 °C for 10 min) for final extension of the amplified DNA (Eppendorf Mastercycler gradient, Hamburg, Germany) [20].

#### 2.1.5. PCR-amplified product purification and sequencing

The PCR products were purified and directly sequenced using a 16-capillary 3100 Genetic Analyser (Applied Biosystem, Foster City, CA, USA). A BigDye® Terminator v3.1 Cycle Sequencing Kit (Applied Biosystems, Foster City, CA, USA) was used according to the manufacturer's protocol.

#### 2.1.6. Data analysis

The obtained *gDNA* sequence was entered into BLAST. The NCBI Nucleotide Collection (nr/nt) database was selected and the query was submitted. Sequences producing significant alignment were identified, and the top 10 hit blast was selected for Multiple Sequencing Alignment (MSA) using Clustal Omega [21].

#### 2.1.7. Phylogenetic analysis

Using the neighbouring-joining (NJ) in Molecular Evolutionary Genetic Analysis (MEGA-X), the evolutionary distance (*K_nuc_*) of identical fungal species was calculated and a phylogenetic tree was generated. The species with closest *K_nuc_* were considered the same species [21].

### 2.2. Submerged-liquid fermentation

*G. lucidum* QRS 5120 was subjected to batch fermentation in a 500-mL Erlenmeyer flask using the optimal media compositions and growth parameters (Table 1).

**Table 1.** Experimental range and levels of the independent variables.

Independent variables	Range and levels		
	−1	0	1
Initial pH	4	5	6
Glucose (g/L)	10	30	50
Agitation (rpm)	90	100	110

#### 2.2.1. Optimisation of media (initial pH, glucose and agitation) using RSM

Based on the preliminary studies, initial pH was shown to have a high significance for the responses (mycelial biomass, EPS production and IPS production) [data not shown]. The media composition of seed culture in the shake flask were constant at (g/L): yeast extract 1 (Oxoid no. LP0021, Dardilly, France), KH_2_PO_4_ 0.5 (Bendosen Laboratory Chemicals no. C0637, Bendosen, Norway), K_2_HPO_4_ 0.5 (Bendosen Laboratory Chemicals no. C0680-2296192, Bendosen, Norway), MgSO_4_ 0.5 (Bendosen Laboratory Chemicals no. C0481, Bendosen, Norway), and NH_4_Cl_2_ 4 (Bendosen Laboratory Chemicals no. C0055, Bendosen, Norway), unless otherwise stated [21]. To optimise the mycelium biomass, EPS and IPS production, CCD was used. The levels and range of the variables for this study are shown in Table 1. The lowest level of variables was initial pH 4; starting glucose concentration = 10 g/L; agitation rate = 90 rpm and the highest level of variables were initial pH 6; starting glucose concentration = 50 g/L; agitation rate = 110 rpm.

To analyse the impact of factors and their interaction, an empirical model was established based on a second-order quadratic model for the responses, as shown in Eq 1:

$$Y=b_{0}^{\prime }+\overset{n}{\underset{i=1}{\sum }}b_{i}X_{i}+\overset{n}{\underset{i=1}{\sum }}b_{ii}X_{i}^{2}+\overset{n}{\underset{i=1}{\sum }}\overset{n}{\underset{j>1}{\sum }}b_{ij}X_{i}X_{j}$$ (1)

where *Y* is the predicted response, $b_{0}^{\prime }$ is the constant coefficient, *b_i_* is the linear coefficient, *b_ij_* is the interaction coefficient, *b_ii_* is the quadratic coefficient and *X_i_X_j_* are the coded values.

### 2.3. Analytical methods

#### 2.3.1. Mycelium biomass

After the tenth day of fermentation, a 50 mL of sample was filtered using a Buchner funnel filter and the mycelial biomass (Figure 1E) was washed three times with distilled water. The filtered mycelial was dried in a food dehydrator at 35 °C to a constant weight. The mycelial biomass was calculated by subtracting the weight of pre-dried filter paper before filtering from the weight of filter paper with mycelial biomass. To obtain the concentration of mycelial biomass, the value obtained from the subtraction was multiplied by the dilution factor [12].

#### 2.3.2. Exopolysaccharide (EPS)

The supernatant obtained from filtering the mycelial biomass (Section 2.3.1) was mixed with ethanol (95%; 4 volumes), stirred and maintained at 4 °C overnight. The mixture was then transferred to a pre-weighed 50 mL Falcon tube and centrifuged (10,000 rpm, 15 min). The supernatant was discarded upon centrifuging and the pellet was placed in a food dehydrator at the lowest temperature until a constant weight was achieved. Next, the EPS yield was estimated by multiplying the dilution factor by the EPS weight [12].

#### 2.3.3. Intracellular polysaccharide (IPS)

After weighing, the filtered mycelial biomass (Section 2.3.1) was mixed with distilled water (10 volumes). Then, the mixture was sterilised (121 °C, 30 min, 15 psi) in an autoclave and the mixture was filtered to obtain the supernatant, mixed with ethanol (95%; 4 volumes), stirred and maintained at 4 °C overnight. Next, the mixture was transferred to a pre-weighed 50 mL Falcon tube and centrifuged (10,000 rpm, 15 min). The supernatant was discarded, and the pellet was placed in a food dehydrator at the lowest temperature until a constant weight was achieved. The IPS yield was estimated by multiplying the dilution factor by the IPS weight [14].

## 3. Results and discussion

### 3.1. Molecular characterisation

#### 3.1.1. Gel electrophoresis

**Figure 2.**
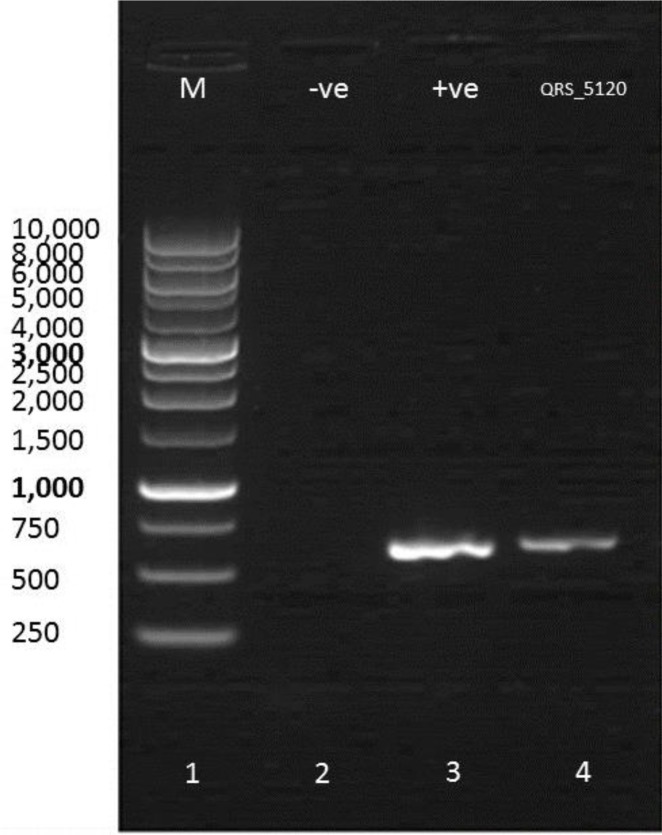
Agarose gel electrophoresis of DNA isolated from *Ganoderma lucidum* mycelium. Lane 1 corresponds to 10 kb marker. Lane 2 corresponds to negative control (−ve), Lane 3 corresponds to positive control (+ve) and Lane 4 corresponds to the sample (QRS_5120).

Molecular identification of a wild fungal sample is important to determine the species of the sample [22]. Thus, molecular identification was performed on wild *G. lucidum*. The base pairs of wild *G. lucidum* were estimated using agarose gel electrophoresis under UV light (Figure 2). The marker (Lane 1) represented the standard curve, and the base pairs of QRS 5120 were estimated to be 637 bp.

#### 3.1.2. Phylogenetic tree

**Figure 3.**
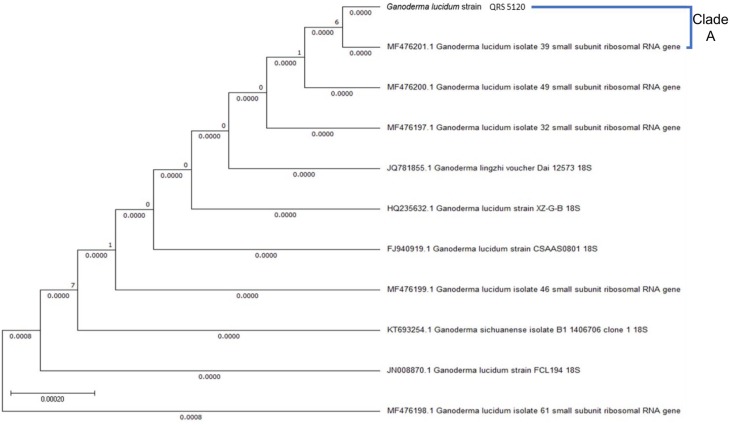
Phylogenetic tree of *Ganoderma lucidum* strain QRS_5120 with evolutionary distance. Bar = 0.00020.

Upon sequencing of the product, it was aligned with the top-10 related species as retrieved from NCBI BLAST. Based on the BLAST reference databases, QRS 5120 was found to be 99% similar to *Ganoderma* sp. Detailed phylogenetic analyses (Figure 3) showed the evolutionary distance (*K_nuc_*) values. Clade A showed that *G. lucidum* QRS 5120 was closely related to *G.* *lucidum* isolate 39s compared with *G.* *lucidum* isolate 49s.

### 3.2. Optimization

Using RSM, the effect of initial pH, starting glucose concentration and agitation rate on the biomass, EPS and IPS production was investigated. CCD design, the level of each variable and the responses are shown in Table 2. In total, twenty experiments were designated by CCD, where the coefficients were evaluated using non-linear regression analysis. To estimate the significance of the model coefficient, analysis of variance (ANOVA) was used. The significance of each coefficient was indicated by *p* < 0.05.

**Table 2.** Experimental design matrix using RSM with CCD and responses for the mycelial biomass (DCW), EPS and IPS production from the mycelium of *G. lucidum* strain QRS 5120.

Run No.	Variables				Responses					
	Initial pH	Glucose (g/L)	Agitation (rpm)		Biomass (DCW g/L)		EPS (g/L)		IPS (g/L)	
					Actual	Predicted	Actual	Predicted	Actual	Predicted
1	4	10	90		4.6	4.52	2.2	2.31	1.2	1.28
2	6	50	110		3.8	3.92	1.2	1.13	0.2	0.14
3	6	50	90		3.1	2.97	0.9	0.91	0.2	0.25
4	5	30	100		4.1	4.12	1.1	1.31	0.8	0.85
5	5	30	90		3.9	3.6	1.3	1.15	0.7	0.73
6	5	50	100		3.8	3.96	1	0.91	0.9	0.83
7	5	30	100		4.2	4.12	1.4	1.31	0.9	0.85
8	4	50	110		5.1	4.79	2.1	2.18	1.4	1.53
9	5	30	100		3.8	4.12	1	1.31	0.9	0.85
10	4	50	90		4.9	5.04	1.9	1.96	1.5	1.44
11	5	30	100		3.9	4.12	1.3	1.31	0.7	0.85
12	5	30	110		4.2	4.3	1.4	1.37	0.9	0.77
13	5	30	100		4.2	4.12	1.5	1.31	0.8	0.85
14	5	30	100		4.1	4.12	1.2	1.31	0.8	0.85
15	4	30	100		5.2	5.26	2.9	2.61	1.7	1.57
16	6	10	110		4.1	4	1.2	1.18	0.1	0.18
17	6	30	100		4	3.74	1.3	1.41	0.3	0.33
18	4	10	110		4.8	4.97	2.5	2.53	1.5	1.47
19	5	10	100		4.1	3.74	1.2	1.11	0.8	0.77
20	6	10	90		2	2.35	1	0.96	0.3	0.19

#### 3.2.1. Optimization of mycelium biomass production

The ANOVA for mycelium biomass production is shown in Table 3. The predicted coefficient determination indicates that 91.47% (*R^2^* = 0.9147) of the variability in the response can be explained using this model. The model is significant (*p* < 0.005). The adjusted coefficient determination value (Adj. *R^2^* = 0.84) implies the significance of the model and is in reasonable agreement with the predicted *R^2^* value. By considering the significant terms, the model, in terms of actual variables of biomass, was regressed and is expressed by Eq 2.

$$Biomass=9.515227273-7.661136364\;\times pH+0.126295455\times Glucose+0.238522727\times Agitation+0.386363636\times pH^{2}-0.000659091\times Glucose^{2}-0.001636364\times Agitation^{2}+0.00125\times pH\times Glucose+0.03\times pH\times Agitation-0.00875\times Glucose\times Agitation$$ (2)

**Table 3.** Analysis of variance (ANOVA) for the experimental results of the CCD quadratic model for biomass from the mycelium of *G. lucidum* strain QRS_5120.

Source	Sum of Squares	DF	Mean Square	*F* Value	Prob > *F*	
Model	8.588136364	9	0.954237374	11.90767001	0.0003*	significant
A: pH	5.776	1	5.776	72.07714124	<0.0001*	significant
B: Glucose	0.121	1	0.121	1.509926262	0.2473	
C: Agitation	1.225	1	1.225	15.28644356	0.0029*	significant
A^2^	0.410511364	1	0.410511364	5.122660238	0.0471*	significant
B^2^	0.191136364	1	0.191136364	2.385138968	0.1535	
C^2^	0.073636364	1	0.073636364	0.918888259	0.3604	
AB	0.005	1	0.005	0.062393647	0.8078	
AC	0.72	1	0.72	8.984685196	0.0134*	significant
BC	0.245	1	0.245	3.057288712	0.1109	
Residual	0.801363636	10	0.080136364			
Lack of Fit	0.666363636	5	0.133272727	4.936026936	0.0522	not significant
Pure Error	0.135	5	0.027			
Cor Total	9.3895	19				

Std. Dev. = 0.28308366896796		*R^2^* = 0.91465321514845		Adeq Precision = 14.52399050576		
Mean = 4.095		Adjusted *R^2^* = 0.83784110878205				

* Significant value.

**Figure 4.**
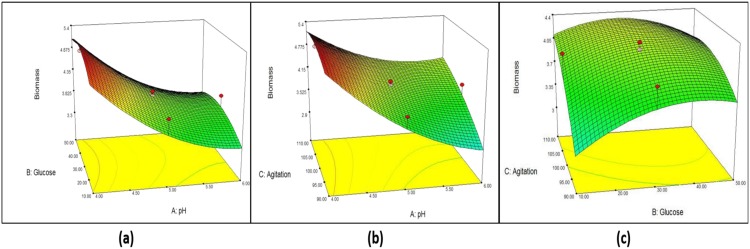
Response surface curve (3D plot) of mycelium biomass from *G. lucidum* strain QRS 5120 showing the interaction between (a) pH and glucose, (b) pH and agitation, (c) Glucose and agitation.

From the model, initial pH (A) shows the strongest effect (*p* < 0.0001) on biomass, while agitation (C) shows a significant effect at *p* < 0.05. Both quadratic terms of initial pH (AA) and initial pH and agitation (AC) show significance effect at *p* < 0.05 on the yield of mycelium biomass. However, negative effects are shown by glucose (B) and quadratic terms (B^2^, C^2^, AB, and BC). Figure 4 shows the combined effect of initial pH, glucose concentration and agitation in three-dimensional (3D) plots. One factor is at the optimum level and the other two factors are within experimental range. Figure 4a shows the effect of initial pH (A) and starting glucose concentration (B), Figure 4b shows the effect of A and agitation rate (C), and Figure 4c shows the effect of B and C on biomass production. From Figure 4a,b, it is clear that increasing the initial pH leads to a decrease in the mycelium biomass, while agitation at all rates shows that high mycelium biomass production and starting glucose concentration is normally distributed. The maximum mycelium biomass obtained was at initial pH 4, glucose concentration 26.5 g/L and 100 rpm. From Figure 4c, no significance effect of B and C on mycelium biomass production was observed.

#### 3.2.2. Optimization of EPS production

The ANOVA of EPS production is shown in Table 4. The predicted coefficient determination indicates that 93.58% (*R^2^* = 0.9358) of the variability in the response can be explained using this model. The model was significant (*p* < 0.005). The adjusted coefficient determination value (Adj. *R^2^* = 0.8780) implies the significance of the model and is in reasonable agreement with the predicted *R^2^* value. By considering the significant terms, the model, in terms of actual variables of biomass, was regressed and is expressed in Eq 3.

$$EPS=15.17090909-7.667045455\;\times pH+0.021931818\times Glucose+0.120090909\times Agitation+0.695454545\times pH^{2}-0.000761364\times Glucose^{2}-0.000545455\times Agitation^{2}+0.00375\times pH\times Glucose+4.76916e^{-16}\times pH\times Agitation-3.80257e^{-18}\times Glucose\times Agitation$$ (3)

**Table 4.** Analysis of variance (ANOVA) for the experimental results of the CCD quadratic model for EPS production the mycelium of *G. lucidum* strain QRS_5120.

Source	Sum of Squares	DF	Mean Square	*F* Value	Prob > *F*	
Model	5.364181818	9	0.596020202	16.20420717	<0.0001*	significant
A: pH	3.6	1	3.6	97.8744439	<0.0001*	significant
B: Glucose	0.1	1	0.1	2.718734553	0.1302	
C: Agitation	0.121	1	0.121	3.289668809	0.0998	
A^2^	1.330056818	1	1.330056818	36.16071429	0.0001*	significant
B^2^	0.255056818	1	0.255056818	6.934317845	0.0250*	significant
C^2^	0.008181818	1	0.008181818	0.222441918	0.6473	
AB	0.045	1	0.045	1.223430549	0.2946	
AC	0	1	0	0	1.0000	
BC	0	1	0	0	1.0000	
Residual	0.367818182	10	0.036781818			
Lack of Fit	0.192818182	5	0.038563636	1.101818182	0.4589	not significant
Pure Error	0.175	5	0.035			
Cor Total	5.732	19				

Std. Dev. = 0.19178586543804		R^2^ = 0.93583074287889		Adeq Precision = 12.53566341056		
Mean = 1.48		Adjusted R^2^ = 0.8780784114699				

* Significant value.

**Figure 5.**
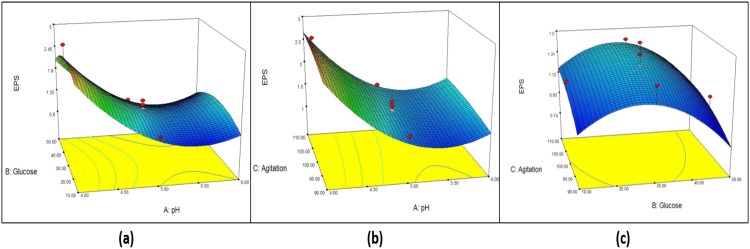
Response surface curve (3D plot) of EPS production from *G. lucidum* strain QRS 5120 showing the interaction between (a) pH and glucose, (b) pH and agitation, (c) Glucose and agitation.

From the model, initial pH (A) shows the strongest effect (*p* < 0.0001) on EPS concentration while both quadratic terms of initial pH (AA) and initial pH and glucose (BB) show a significance effect at *p* < 0.005 and *p* < 0.05, respectively, on EPS production. However, negative effects are shown by glucose (B), agitation (C) and quadratic terms (C^2^, AB, AC and BC). Figure 5 shows the combined effect of initial pH, glucose concentration and agitation in 3D plots. One factor is at the optimum level and the other two factors are within experimental range. Figure 5a shows the effect of initial pH (A) and starting glucose concentration (B), Figure 5b shows the effect of A and agitation rate (C) and Figure 5c shows the effect of B and C on biomass production. From Figure 5a,b, increasing initial pH leads decreased EPS production, agitation at all rates shows high EPS production and starting glucose concentration is normally distributed. The maximum EPS obtained was at initial pH 4, glucose concentration 26.5 g/L and 100 rpm. From Figure 5c, no significance effect of B and C on mycelium biomass production was observed.

#### 3.2.3. Optimization of IPS production

The ANOVA of IPS production is shown in Table 5. The predicted coefficient determination indicates that 96.88% (*R^2^* = 0.9688) of the variability in the response can be explained using this model. The model is highly significant (*p* < 0.0001). The adjusted coefficient determination value (Adj. *R^2^* = 0.9407) implies the significance of the model and is in reasonable agreement with the predicted *R^2^* value. By considering the significant terms, the model, in terms of actual variables of biomass, was regressed and is expressed in Eq 4.

$$IPS=-6.393409091-1.127954545\;\times pH+0.027068182\times Glucose+0.221659091\times Agitation+0.104545455\times pH^{2}-0.000113636\times Glucose^{2}-0.000954545\times Agitation^{2}-0.00125\times pH\times Glucose-0.005\times pH\times Agitation-0.000125\times Glucose\times Agitation$$ (4)

**Table 5.** Analysis of variance (ANOVA) for the experimental results of the CCD quadratic model for IPS production the mycelium of *G. lucidum* strain QRS_5120.

Source	Sum of Squares	DF	Mean Square	*F* Value	Prob > *F*	
Model	3.935181818	9	0.437242424	34.47789725	<0.0001*	significant
A: pH	3.844	1	3.844	303.1111111	<0.0001*	significant
B: Glucose	0.009	1	0.009	0.709677419	0.4192	
C: Agitation	0.004	1	0.004	0.315412186	0.5867	
A^2^	0.030056818	1	0.030056818	2.370071685	0.1547	
B^2^	0.005681818	1	0.005681818	0.448028674	0.5184	
C^2^	0.025056818	1	0.025056818	1.975806452	0.1901	
AB	0.005	1	0.005	0.394265233	0.5441	
AC	0.02	1	0.02	1.577060932	0.2377	
BC	0.005	1	0.005	0.394265233	0.5441	
Residual	0.126818182	10	0.012681818			
Lack of Fit	0.098484848	5	0.01969697	3.475935829	0.0989	not significant
Pure Error	0.028333333	5	0.005666667			
Cor Total	4.062	19				

Std. Dev. = 0.11261357902943		R^2^ = 0.96877937424466		Adeq Precision = 17.969511850411		
Mean = 0.83		Adjusted R^2^ = 0.94068081106486				

* Significant value.

**Figure 6.**
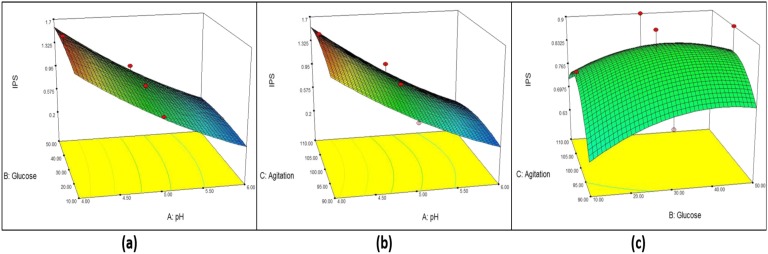
Response surface curve (3D plot) of IPS production from *G. lucidum* strain QRS 5120 showing the interaction between (a) pH and glucose, (b) pH and agitation, (c) Glucose and agitation.

From the model, initial pH (A) shows the strongest effect (*p* < 0.0001) on IPS concentration. However, negative effects are shown by glucose (B), agitation (C) and quadratic terms (A^2^, B^2^, C^2^, AB, AC and BC). Figure 6 shows the combined effect of initial pH, glucose concentration and agitation in 3D plots. One factor is at the optimum level and the other two factors are within experimental range. Figure 6a shows the effect of initial pH (A) and starting glucose concentration (B), Figure 6b shows the effect of A and agitation rate (C) and Figure 6c shows the effect of B and C on biomass production. From Figure 6a,b, it is clear that increasing initial pH leads to decreased IPS production, agitation at all rates shows high IPS production and all concentrations of starting glucose give high IPS concentration. By this, it was concluded that there was no interaction between the factors. The maximum IPS obtained was at initial pH 4, glucose concentration 40.45 g/L and 103 rpm. From Figure 6c, no significance effect of B and C on IPS production was observed.

#### 3.2.4. Verification of the optimized conditions

Table 6 shows the optimised conditions applied to verify the mycelium biomass, EPS and IPS production statistical model. To verify the strength and precision of the model under Eq 2, Eq 3 and Eq 4, various experiments were performed. The mycelium biomass obtained was 5.12 g/L, EPS production under optimized conditions was 2.49 g/L, and IPS production under optimized conditions was 1.52 g/L, which aligns with the predicted values (5.25 g/L, 2.69 g/L and 1.59 g/L, respectively). This shows that the model (Eq 2, Eq 3 and Eq 4) is valid for mycelium biomass, EPS and IPS production.

**Table 6.** Validation of the model with the optimized conditions.

Run	Variables			Response		
	pH	Glucose	Agitation	Biomass (DCW g/L)	EPS (g/L)	IPS (g/L)
Biomass	4.01	32.09	102.45	5.12 ± 0.5	−	−
EPS	4	24.25	110	−	2.49 ± 0.8	−
IPS	4	40.43	103	−	−	1.52 ± 0.4
Biomass + EPS	4	24.75	107.58	5.11 ± 0.4	2.57 ± 0.7	−
Biomass + IPS	4	40.45	102.95	5.13 ± 0.5	−	1.57 ± 0.3
EPS + IPS	4	26.5	105.92	−	2.62 ± 0.4	1.52 ± 0.6
Biomass + EPS + IPS	4	26.5	100	5.19 ± 0.6	2.64 ± 0.6	1.52 ± 0.4

### 3.3. Comparison of the current study with the literature

The current statistical optimization approach to determine the preeminent parameters for obtaining efficient biomass, EPS and IPS production using *G. lucidum* in controlled shake-flask fermentation is shown in Table 7. As reported, only two studies utilizing *G. lucidum* for this purpose have previously been reported, utilizing only EPS and biomass production. ChangTsai and Houng [23] stated that mycelium formation and polysaccharide production were markedly improved by cultivation in optimal medium under optimal operating conditions. YuanChi and Zhang [24] also supported this observation by stating that optimal media markedly increases EPS production and mycelium formation. The process described in the current study was more efficient in producing biomass and EPS concentration compared with the results of YuanChi and Zhang [24] and ChangTsai and Houng [23], with the addition of IPS. The optimized key parameters reported in this study are the most up-to-date for *G. lucidum* using RSM on initial pH, glucose concentration and agitation for enhancing the production of biomass, EPS and IPS. The current optimized method can therefore be applied to achieve a combination of lower biomass with higher EPS and IPS in specialized bioreactors.

**Table 7.** Comparison of *Ganoderma lucidum* optimization using submerged-liquid fermentation with the literature.

Optimization method	Cultivation mode	Initial pH	Glucose concentration (g/L)	Agitation (rpm)	EPS (g/L)	IPS (g/L)	Biomass (g/L)	Reference
Response surface methodology	Shake Flask	4	26.5	100	2.64	1.52	5.19	Current study
Taguchi's orthogonal array	Shake Flask	6.5	12.1	160	0.420	NA	18.70	[23]
Orthogonal matrix	Shake Flask	−	50	150	1.723	NA	7.235	[24]

*NA = Not available, EPS = Exopolysaccharide, IPS = Intracellular polysaccharide.

## 4. Conclusions

The wild-cultivated Malaysian medicinal mushroom *G. lucidum* strain QRS 5120 was successfully identified using morphological and biomolecular methods. Using response surface methodology in a submerged-liquid fermentation, maximum mycelial biomass (5.19 ± 0.6 g/L) and EPS (2.64 ± 0.6 g/L) were obtained under optimized conditions of initial pH 4, 26.52 g/L glucose concentration and 103 rpm. On the other hand, maximum IPS production (1.57 ± 0.3 g/L) was obtained under optimized conditions of initial pH 4, glucose concentration 40.45 g/L and agitation 103 rpm. In addition, in order to maximise all three responses together, i.e. biomass, EPS and IPS (5.19 g/L, 2.64 g/L and 1.52 g/L respectively), the optimised conditions were initial pH 4, glucose concentration at 26.5 g/L and 100 rpm. It was found that by using the optimised conditions, the biomass, EPS and IPS production could be maximized. The initial pH could significantly affect biomass, EPS and IPS production in comparison to glucose concentration and agitation rate. The current work is suitable to be applied for other type of fungal fermentation for efficient EPS, IPS and biomass production.
